# Is the Incidence Trend of Heparin-Induced Thrombocytopenia Decreased by the Increased Use of Low-Molecular-Weight-Heparin?

**DOI:** 10.4084/MJHID.2015.029

**Published:** 2015-04-20

**Authors:** Fahad A S Al-Eidan

**Affiliations:** College of Medicine, King Saud bin Abdulaziz University for Health Sciences, Kingdom of Saudi Arabia

## Abstract

**Background:**

The increasing trend of using low-molecular-weight-heparin (LMWH) versus unfractionated heparin (UFH) in hospitalized adult patients is raising concerns about the incidence of heparin-induced thrombocytopenia (HIT).

**Method:**

A retrospective study analyzed the requests for heparin-induced antibodies by enzyme-linked immunosorbent assay (ELISA) among adult hospitalized patients during the period from January 2011 to December 2013. These patients received either UFH or LMWH for prevention or therapeutic indications. Those with positive immune-mediated HIT were identified and considered as case patients.

**Result:**

The usage of LMWH and UFH and development of HIT was determined during the study period. The incidence of HIT in patients receiving UFH and those receiving LMWH was 4.09 per thousand patients and 0.48 per thousand patients, respectively, (p<0.0001) with an overall incidence of 2.49 per thousand patients.

**Conclusion:**

The increased trend of using LMWH over UFH among hospitalized adult patients was observed and can be said to contribute to the diminished overall incidence of HIT.

## Introduction

Venous thromboembolism (VTE) is the most common preventable cause of hospital morbidity and mortality. A preventative pharmacological agent is recommended for all hospitalized patients at risk of developing VTE. Low-molecular-weight-heparin (LMWH) and unfractionated heparin (UFH) are widely used and cost effective VTE prevention agents.^1^,^2^ However, heparin-induced thrombocytopenia (HIT) is an immune-mediated, potentially life-threatening adverse effect of heparin therapy.^3^,^4^ Heparin can induce immunoglobulin G (IgG) antibodies production against itself and platelet factor 4 (PF4); the antibodies stimulate platelets and endothelial cells, resulting in an excess production of thrombin, inducing thrombocytopenia and thromboembolic events.^5^–^7^ HIT occurs in approximately 3% of patients who receive UFH and approximately 0.2% of patients who receive LMWH.^4^,^8^–^11^

HIT is clinically diagnosed by a drop in platelet count to less than 100×109/L or a 50% decrease in platelets after the initiation of heparin therapy with no apparent explanation other than HIT.^12^ A positive laboratory test for HIT antibodies supports this clinical diagnosis. The development of HIT can be either; delayed-onset, typically 5 to14 days after the initial administering of heparin, or rapid-onset, occurring soon after the re-administering of heparin to a patient with prior heparin exposure and HIT antibodies.^7^,^13^,^14^

Heparin exposure has a unique HIT complication that is characterized by a defined thrombocytopenia and immune-mediated platelet activation that can lead to thrombin over-production and increase the chance of developing VTE in the majority of patients. This can lead to life-threatening complications.^15^,^16^

A retrospective database analysis was performed on the annual incidence of HIT at a single teaching center. We assessed the effect of prescribing UFH and LMWH, with additional analysis of the annual lab requests for HIT antibodies and confirmed positive HIT tests

## Methods

Data from The King Abdulaziz Medical City, (KAMC) Riyadh, Saudi Arabia, was used to conduct a retrospective study. The following characteristic data were extracted from patient’s medical record: age, gender, admitting services, indication of heparin administration, and type of heparin.

All patients aged at least 18 years who were admitted to the hospital between January 1, 2011, and December 31, 2013, and who received LMWH or UFH at prevention or therapeutic doses during admission, were reviewed. Enoxaparin was the only LMWH on KAMC Formulary during the study period. First admission in which the diagnosis of HIT occurred was only considered in patients with multiple readmissions. The main clinical suspicion parameter was the platelet count, determined as follows: pretreatment platelet count at baseline, and then every 2 to 3 days from commencing UFH or LMWH administration for first two weeks.

Thrombocytopenia was defined as a platelet count fall of ≥50% from a baseline that was apparent by HIT diagnosis based on clinical probability which routinely evaluated according to the 4Ts score system by the hematology services. The origin of thrombocytopenia was confirmed by the detection of serum heparin-induced antibodies, using a commercial enzyme-linked immunosorbent assay (ELISA) for IgG, IgA, and IgM class antibodies (Asserachrom HPIA, Diagnostica Stago, Asniere sur Seine, France).^1^,^24^,^25^ All HIT positive patients were diagnosed and labeled by hematology services. ELISA was performed in the KAMC hematology laboratory according to manufacturer’s procedures. HIT results expressed in optical density (OD) units and a value of >0.4 was considered to be positive according to the manufacturer’s range. Exclusion criteria were as follows: (1) patients who developed HIT or thrombocytopenia before the study period and still in the hospital during the study period. (2) Heparin or its derivatives given after the thrombocytopenia occurred. (3) Thrombocytopenia explained by other conditions such as a chemotherapeutic agent being administered. From this data, the annual number of patients who received UFH or LMWH for prevention or therapeutic indications and the total number of heparin-induced antibody assays performed over the study period was determined.

Identified HIT patients were divided into three groups: (1) patients receiving LMWH; (2) patients receiving UFH; and (3) total number of patients receiving LMWH and UFH. The incidence of HIT was determined for each group, and the HIT incidence trend was also determined over the study period. The relative risk was calculated by comparing patients exposed to UFH and those exposed to LMWH for prevention or therapeutic indications.

### Statistical analysis

Data was summarized as means (S.D) or proportions. Cumulative incidence rate and 95% confidence interval (CI) was calculated per one thousand patients. Comparison between incidence rates was conducted using a chi-square test. All tests were two-sided and a P value < 0.05 was considered significant. The STATA statistical software (STATACORP, TX, USA, version 11) was used to carry out the statistical analysis.

## Results

The main clinical, demographic characteristics of the 116 patients who developed HIT including sex, age, indication, admitting hospital department, and laboratory results are summarized in Table 1.

Sixty-seven patients were male, and 49 were female. The mean (±SD) age was 64 (±16). UFH for prevention indication (65.5%) was used more frequently than LMWH. The mean (±SD) platelet nadir was 82 (±18). The majority of HIT patients (62.1%) had a high clinical the 4Ts score. ELISA assays were reported and classified by OD values. HIT patients were associated with a decreased albumin level (Mean ±SD; 25±7).

Table 2 describes the annual development of HIT in our data from January 2011 to December 2013. In patients receiving UFH and those receiving LMWH, the annual incidence rate of HIT per one thousand patients was 4.42 and 0.46 in 2011 (P<0.0001); 4.19 and 0.47 in 2012 (P<0.0001); 3.48 and 0.50 in 2013 (P<0.0001), respectively, with an over 3-year incidence of 4.09 and 0.48 (P<0.0001) respectively. The patients who received UFH were 8.5 times more likely to develop HIT than those who received LMWH.

**Figure 1:**
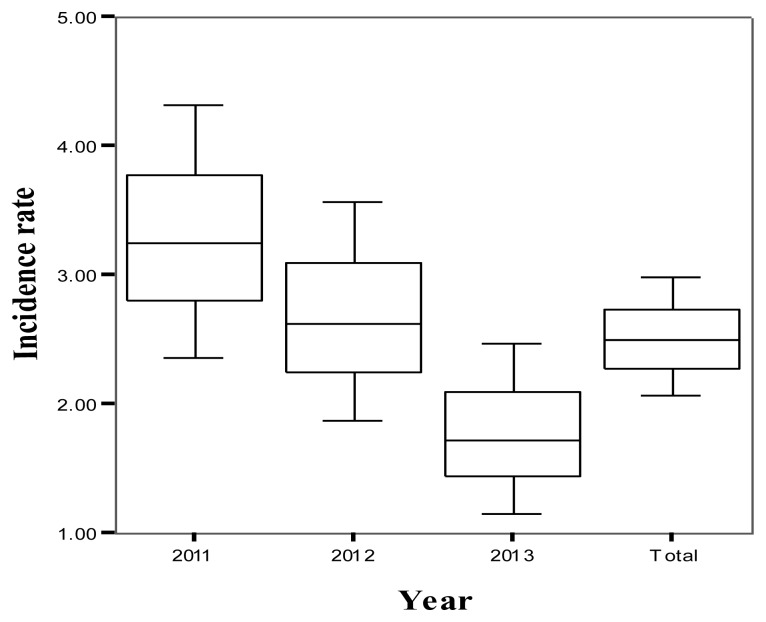
Trends in the incidence rates of HIT per 1000 patients from 2011 to 2013 and the total over a three-year study period incidence rate. Each bar represents incidence rate and confidence intervals.

A decrease in the total annual incidence rate of HIT, UFH and LMWH, was observed: 3.24 in 2011, 2.62 in 2012 and 1.72 in 2013 (Figure 1). The difference in the incidence of HIT between 2011 and 2012 was not statistically significant (difference =0.63, 95%CI −0.67 to 1.92, P=0.32). Similarly the difference between 2012 and 2013 was not statistically significant (difference=0.90, 95%CI −0.19 to 1.99, P=0.08). However the difference between the incidence in 2011 and 2013 was statistically significant (difference=1.53, 95%CI 0.36 to 2.71, P=0.006).

**Figure 2:**
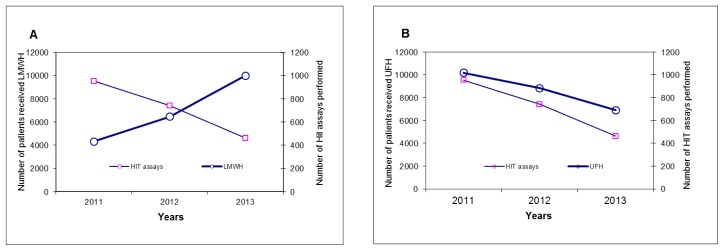
Graph ‘**A**’ shows a correlation between the annual number of patients receiving low molecular weight heparin (LMWH) and annual number of heparin-induced thrombocytopenia (HIT) assays performed. ‘**B**’ shows the correlation between annual number of patients receiving unfractionated heparin (UFH) and annual numbers of HIT assays.

The annual number of hospitalized patients who received heparin (UFH and LMWH) and the number of HIT assays performed with HIT test results are summarized in Table 3. Briefly, the number of patients who received UFH decreased from 10,175 patients (70%) in 2011 to 6,890 (40%) in 2014, while the number of patients who received LMWH increased from 4,309 patients (30%) in 2011 to 9,989 (59) in 2014. The total number of patients who received heparin (UFH or LMWH) increased from 14,484 patients in 2011 to 16,879 in 2014. However, the total annual HIT assays performed decreased by 48 % from 953 tests in 2011 to 462 in 2014. The annual number of patients receiving LMWH inversely correlated with annual number of HIT assays performed (Figure 2A), while the annual number of patients receiving UFH correlated very closely with the annual numbers of HIT assays performed (Figure 2B).

## Discussion

To our knowledge, this is the first study to describe the cumulative incidence rate of HIT in an adult hospitalized Saudi population. This study showed that the cumulative incidence rate of HIT when using UFH was 4.09 per 1,000 patients, and the incidence when using LMWH was 0.48, with the overall cumulative incidence being 2.49. Our incidence is consistent with that of most previously published figures.^4^,^8^,^17^,^18^ Similarly, other authors found that LMWH is associated with lower risk of HIT than UFH.^10^,^11^

A trend towards increasing the annual use of LMWH vs. UFH was observed between 2011 and 2013 (Table 3). Interestingly, the increased use of LMWH during the study period showed a direct impact as the annual incidence rate decreased significantly between 2011 and 2013. (3.24 per one thousand patients vs. 1.72, respectively with P=0.006).

Despite of the annual increase in the number of patients receiving heparin (UFH and LMWH) during the study period, there was a significant decrease in the annual number of requests for heparin-induced antibodies by ELISA between 2011 and 2013. This correlation may be due to the increased use of LMWH over UFH and its low effect on platelet count and may have contributed to our finding. Zhou et al (2012), in a study conducted between 2005 and 2009, observed that despite a doubling in the number of patients receiving pharmacoprophylaxis with heparin, there was no significant increase in the number of consultations for thrombocytopenia, the number of requests for HIT tests, the number of positive HIT test results, or the number of HIT diagnoses. In this period there was a significant increase of proportion of patients treated with LMWH The number of cases of HIT was low and represented < 0.1% of patients exposed to heparin.^19^ Therefore, many authors in previous studies have suggested LMWH as a preferred agent, despite the higher cost per dose, due to a low risk of thrombocytopenia and HIT.^10^,^11^,^20^–^23^

The current study has several limitations such as its retrospective nature, which raises concerns about measured and unmeasured bias that may lead to misclassifying patients who have HIT or not. A further limitation is that a PF4-dependent ELISA was used to detect antibodies against PF4/heparin to confirm the HIT diagnosis. Although functional assays are more specific for detecting HIT antibodies than PF4-dependent ELISA,^1^,^2^,^24^ an ELISA assay is often used because of its rapid performance, and lower cost compared with a functional test. However, functional tests were not performed in the center where the study was conducted which may have affected the HIT result identification. In addition, outcomes such as complications and survival rate have not been possible to identify accurately, nor was a specific population group targeted in the study. Finally, concurrent agents that may contribute to the development of thrombocytopenia were not considered except chemotherapy.

## Conclusion

In this three-year study period, we identified a decreasing incidence rate of HIT in hospitalized adult patients that may be attributed to the increasing use of LMWH over UFH.

## Figures and Tables

**Table 1:** Main demographic characteristics of the study group.

No. Of patients	116
Age, y, mean ±SD	64±16
Sex	
Male (%)	67 (58)
Female (%)	49 (42)
Indication for UFH/LMWH therapy	
Prevention	76 (65.5)
Therapeutic	40 (34.5)
Admitting Departments	
Cardiac Science	34 (29.3)
Surgery (GS, Orthopedic, Vascular, etc.)	32 (27.6)
Intensive care	25 (21.5)
Internal Medicine	14 (12.1)
Oncology/Hematology	8 (6.9)
Hepatobiliary Science	3 (2.6)
4Ts score	
Low (0–3)	0 (0)
Intermediate (4–5)	44 (37.9)
High (6–8)	72 (62.1)
Laboratory	
Platelet count, mean ±SD (Normal range: 150–400 ×10^9^/L)	
Before heparin exposure	327 (±108)
After heparin exposure	82 (±29)
ELISA optical density	
>0.4–0.99	4(3.5)
1.0–1.99	61 (52.6)
≥2.0	51 (43.9)
Albumin level before HIT, mean ±SD (Normal range: 35–50 g/L)	25 (±7)

ELISA, enzyme-linked immunosorbent assay; HIT, Heparin-induced thrombocytopenia; LMWH, low molecular weight heparin; UFH, unfractionated heparin.

**Table 2:** Annual incidence rates of HIT per one thousand patients.

	Heparin		
		
Year	UFH	LMWH	P value
2011	4.42(3.27–5.87)	0.46(0.08–1.53)	<0.0001
2012	4.19(3.00–5.72)	0.47(0.12–1.27)	<0.0001
2013	3.48(2.28–5.10)	0.50(0.18–1.11)	<0.0001
Total	4.09 (3.35–4.95)	0.48 (0.23–0.89)	<0.0001

Data is presented as incidence rate (95% confidence interval). HIT, Heparin-induced thrombocytopenia; LMWH, low molecular weight heparin; UFH, unfractionated heparin.

**Table 3:** Annual number of hospitalized patients who received heparin and number of HIT assays performed with HIT test results.

	2011	2012	2013	Total
Heparin				
No. of patients receiving UFH	10175 (70)	8825 (58)	6890 (41)	25890 (56)
No. of patients receiving LMWH	4309 (30)	6450 (42)	9989 (59)	20748 (44)
Total No. of patients received UFH and LMWH	14484	15275	16879	46638

HIT assay				
Positive HIT assays	47 (5)	40 (5.4)	29 (6.3)	116 (5.6)
Negative HIT assays	906 (95)	701 (94.6)	433 (93.7)	1953 (94.4)
Total HIT assays performed	953	741	462	2069

Data is presented as No. (%). HIT, Heparin-induced thrombocytopenia; LMWH, low molecular weight heparin; UFH, unfractionated heparin.
